# The impact of career planning and self-concept on employability among university students: exploring the mediating role of learning attitudes

**DOI:** 10.3389/fpsyg.2026.1818002

**Published:** 2026-06-19

**Authors:** Yu Sun, Qian Liu, Xiaona Liu

**Affiliations:** 1 School of Marxism, Hengshui University, Hengshui, Hebei, China; 2 Teacher Education Department, Hengshui University, Hengshui, Hebei, China

**Keywords:** career planning, employability, learning attitude, self-concept, university students

## Abstract

**Background:**

With the rapid development of education, higher education in China has become increasingly accessible and widespread. The number of university graduates is rising every year, leading to a more competitive job market. As a result, enhancing college students’ employability has become an urgent issue. This study explores the mechanisms through which career planning and self-concept affect employability, and examines the mediating role of learning attitude, aiming to provide both theoretical and practical guidance for improving employability.

**Methods:**

Drawing on the Career EDGE model, this study used convenience sampling to survey 735 undergraduates from universities in Hebei Province, China. Data were collected via questionnaires and analyzed using SPSS Statistics 27 and AMOS. Structural equation modeling (SEM) was applied to test the effects of career planning, self-concept, and learning attitude on employability, and to verify the mediating role of learning attitude.

**Results:**

Career planning, self-concept, and learning attitude all showed significant positive effects on employability. Mediation analysis revealed that learning attitude mediated the relationship between career planning and employability, as well as between self-concept and employability.

**Conclusion:**

The findings demonstrate that career planning and self-concept positively influence employability, and that learning attitude serves as an important pathway in this process. Universities should assist students in developing clear career plans and building a positive self-concept to foster active learning attitudes, thereby enhancing their employability.

## 1 Introduction

According to the 2022 China College Graduate Employment Report released by the Central People’s Government of the People’s Republic of China, the number of fresh graduates in 2022 exceeded 10 million for the first time, marking a record high in both total and incremental numbers. At the same time, a survey report by Zhaopin.com showed that the employment rate of college graduates in 2022 was 50.4%, representing a 6.5% decline compared with 2021 (Zhang, 2023). With the continuing expansion of higher education, the number of college graduates is expected to keep increasing. Coupled with the downward pressure on economic growth, the intersection of a persistently high graduate population and ongoing economic restructuring is bound to create more complex employment challenges (Zhao, 2022). In practice, structural mismatches have become a key issue: many graduates are not unable to find jobs, but rather unable to find satisfactory ones. This reflects the widespread problem of low employment quality (Dai and Wang, 2014). Improving the quality of graduate employment depends on enhancing their employability (Zhao, 2019). Graduate employability has attracted increasing attention from scholars in the fields of higher education and human capital (Guo and Xie, 2022). Employment is a critical issue for the nation’s economy and people’s livelihoods, as it concerns both national stability and individuals’ pursuit of a better life (Zhang, 2023).

Employability is a key factor for maintaining sustainable employment in today’s unstable job market. It also reflects an individual’s flexibility—the ability to be adequately prepared to face the current complex and rapidly changing employment environment. Through learning in a professional field, individuals can acquire the skills needed to secure employment, maintain competitiveness, and continuously develop professional skills (Donald et al., 2018). According to the Career EDGE model of employability proposed by Dacre and Sewell (2007), university students seeking to develop their employability must first enhance five core elements: career development learning, work and life experience, subject-specific knowledge and skills, generic skills, and emotional intelligence (first tier). They must then engage in ongoing reflection and evaluation of these experiences (second tier). This process promotes the development of higher-level attributes such as self-esteem, self-confidence, and self-efficacy, which are essential for building employability (Huang, 2014).

Career planning refers to the process in which individuals set reasonable plans for their future careers to achieve their ideal goals, as well as the strategies and methods they adopt to realize these plans (Gould, 1979; Koen et al., 2013). Career planning is not only part of the “career development learning” component in the Career EDGE model of employability, but also falls under the generic skills dimension, which includes planning, coordination, organization, and management abilities—the first tier in enhancing employability (Dacre and Sewell, 2007). Research indicates that to improve their employability, university students should clearly define their career goals and continuously enrich and improve themselves with career planning as a guiding principle. This involves exploring their potential, enhancing professional qualities, and preparing for future career development to strengthen their competitiveness in the job market (Shury, 2017). Therefore, career planning plays a positive and significant role in promoting students’ employability (Healy et al., 2022).

Self-concept refers to an individual’s overall view and perception of themselves, encompassing their beliefs, positions, and emotions, as well as their self-descriptions and self-definitions regarding physical, psychological, and social characteristics, and personal abilities (Preckel et al., 2013). Within the Career EDGE model of employability, self-concept is part of the first-tier element of emotional intelligence and also falls under the reflection and evaluation stage. It plays an important role in the development of employability (McArdle et al., 2007). Tentama and Abdillah (2019) found through student interviews that the most influential factors affecting employability are academic achievement and self-concept. Similarly, Kim et al. (2015) reported that having a strong and positive self-concept within a specific framework can enhance an individual’s employability, while a low self-concept tends to be associated with lower employability. Therefore, self-concept plays a vital role in improving employability (Tentama and Jayanti, 2019).

According to the Career EDGE model of employability, the second tier is reflection and evaluation. While it is important to provide students with opportunities to acquire the essential knowledge, skills, understanding, and attributes of the first tier, it is equally important to offer opportunities for reflection and evaluation of their learning experiences. Without such opportunities, students cannot fully assess how far they have progressed in developing their employability, nor can they identify the steps needed to further enhance it (Dacre and Sewell, 2007). Learning attitude refers to a learner’s positive or negative evaluations and feelings toward learning activities or environments, as well as their readiness or tendency to engage in active or passive learning behaviors (Chi et al., 2007). Learning attitude belongs to the second-tier “reflection and evaluation” component of the Career EDGE model. A positive learning attitude can effectively promote the employability of university students (Fan and Pan, 2022). During their studies, students should continuously reflect on and adjust themselves according to real circumstances. With an active learning attitude-thinking and learning proactively, and expanding their thinking and imagination-their future academic achievements and employability will be stronger (Werner, 1994).

Although research on college students’ employability has received increasing attention in academia, the mechanisms through which self-concept and career planning influence employability have not been fully explored, particularly with regard to systematic and in-depth examination of mediating pathways. Moreover, there is still a lack of theoretical and empirical studies on employability that align with China’s specific context (Guo and Xie, 2022). Therefore, this study, grounded in the Career EDGE model of employability and situated within the Chinese educational context, investigates the interrelationships among career planning, self-concept, learning attitude, and employability, and further examines the potential mediating role of learning attitude. This research helps address gaps in the existing literature while testing the applicability of the Career EDGE model in China, providing a useful reference for strategies to enhance the employability of Chinese university students.

## 2 Theoretical foundation

The Career EDGE employability model essentially builds a three-stage mechanism of employability formation: basic accumulation, reflective transformation, and the generation of psychological resources. This mechanism is not a simple list of different elements. Instead, it shows the dynamic process through which employability moves from resource preparation to the visible expression of ability. Specifically, the first level of the model includes five basic elements: career development learning, work and life experience, subject knowledge and skills, generic skills, and emotional intelligence. These elements represent the external resources and internal traits that individuals obtain. They form the resource input layer for the whole process of employability development. The second level is reflection and evaluation. It is the key stage in which individuals cognitively process their first-level experiences, construct meaning, and make value judgments. Therefore, it plays an important role as the cognitive transformation layer. The third level includes psychological resources such as self-esteem, self-confidence, and self-efficacy. These resources are the final output of employability development. They directly support individuals’ behavior and adaptability in job-seeking situations. This level can therefore be seen as the ability output layer.

The core theoretical value of this model is that it clearly shows that the move from “having resources” to “developing ability” does not happen automatically. It must go through the key process of reflective cognitive processing. In other words, even if individuals have strong career development learning experience, solid subject knowledge and skills, or a high level of emotional intelligence, these factors may not become useful resources by themselves. Without systematic reflection and evaluation of these experiences and traits, individuals may find it difficult to truly internalize them as psychological resources and ability preparation that support employability behavior. This view goes beyond the simple listing of elements in traditional employability research. It provides an important theoretical lens for understanding the dynamic process through which employability is formed.

Based on the above logic of interaction among the three levels, this study further proposes that learning attitude, as an operational carrier of the second level of “reflection and evaluation,” plays a mediating role among career planning, self-concept, and employability. Learning attitude refers to learners’ positive or negative evaluations and feelings toward learning activities or learning environments. It also refers to their tendency or readiness to engage in active or passive learning behaviors (Chi et al., 2007). The core of this concept lies in learners’ evaluative judgment of learning activities and the related readiness for action. This is also the main task of the “reflection and evaluation” level in the Career EDGE model. Based on this, the theoretical logic of this study can be summarized as follows: individuals first need to form positive evaluative judgments about these resources, namely a positive learning attitude. They also need to transform this attitude into behavioral readiness and a tendency to engage in daily learning. Only in this way can employability be substantially improved.

It should be noted that learning attitude should be distinguished from motivation and learning engagement. Motivation answers the question of “why to learn” and refers to the reasons for behavior. In the Career EDGE model, it belongs to self-motivation at the first level. Learning attitude answers the question of “what evaluation one holds toward learning.” It refers to value judgment and a state of behavioral readiness. Learning engagement answers the question of “how to learn.” It refers to the strength of behavior and the level of concentration. It is the stage of action after attitude. The logical relationship among the three concepts can be described as follows: motivation gives direction, learning attitude forms evaluation, learning engagement turns this evaluation into action, and employability is finally produced. This chain clarifies the conceptual boundary of learning attitude. It also strengthens the theoretical reason for viewing learning attitude as an operational carrier of “reflection and evaluation” in the Career EDGE model.

**Figure 1:**
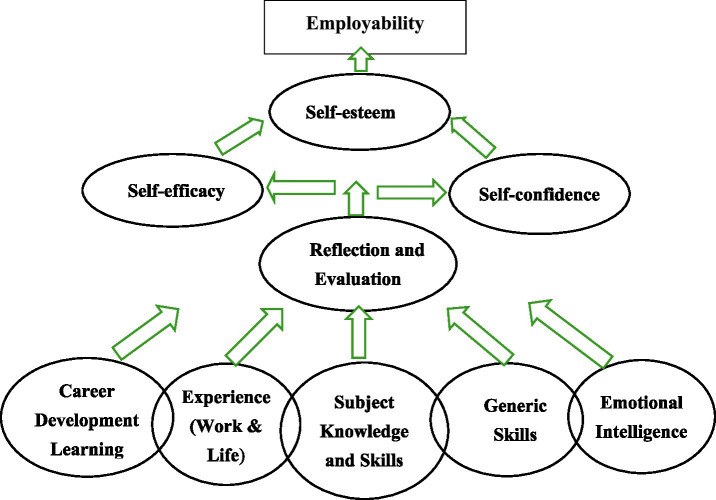
Career EDGE model.

Based on the above analysis, the theoretical model of this study can be summarized as follows: career planning and self-concept, as resource inputs at the first level, are transformed through the mediating role of learning attitude, which represents reflective evaluation and behavioral readiness at the second level. This process finally leads to employability, which reflects psychological resources and behavioral readiness at the third level. This model not only closely reflects the hierarchical interaction mechanism of the Career EDGE model, but also operationalizes the abstract concept of “reflection and evaluation” as the measurable construct of learning attitude. In this way, it builds a methodological link between the theoretical model and empirical research. It also provides a clear and testable theoretical framework for future studies on employability. This model not only faithfully reflects the hierarchical interaction mechanism of the Career EDGE model, but also turns the abstract concept of “reflection and evaluation” into the measurable construct of learning attitude. In this way, it connects the theoretical model with empirical research at the methodological level. It also provides a clear and testable theoretical framework for future research on employability (see Figure 1).

## 3 Literature review and research hypotheses

### 3.1 The impact of career planning on employability

According to the Career EDGE model of employability, career planning falls under the first-tier element of career development learning, as well as the generic skills of planning, coordination, organization, and management. These are the most fundamental factors for enhancing the employability of university students (Dacre and Sewell, 2007). Research has shown that effective career planning is essential for graduates to conduct successful job searches, achieve career satisfaction, and improve their employability-particularly when facing challenging and continuously changing work environments (Ho et al., 2023). By engaging in career planning, students can gain a deeper and more comprehensive understanding of their professional pathways. In the context of current labor market demands, such planning helps them develop effective strategies, advance their career development, and achieve better career outcomes, thereby enhancing their employability (Salisu et al., 2025). Gould (1979) equated career planning with goal setting (Aryee and Debrah, 1993; Wayne et al., 1999), Based on goal-setting theory, employees formulate and implement career strategies to achieve these goals (Tong and Gao, 2022). The effective implementation of such strategies enables them to reach their ultimate career objectives, which in turn strengthens their employability (Clements and Kamau, 2018).

Career construction theory (Ma et al., 2024) suggests that through career exploration, university students can reassess themselves rationally, strengthen the learning of relevant skills, and develop concrete strategies to achieve their goals, thereby enhancing their employability. Similarly, Guo (2021) argued that embedding career planning education throughout the entire undergraduate period—starting from the first year—can awaken students’ career awareness early, help them understand social and job skill requirements in advance, and enable them to plan career paths ahead of time. By purposefully and systematically preparing the professional skills and knowledge needed for future employment, students can develop correct employment concepts and improve their employability. Accordingly, the following hypothesis is proposed:

*H1*: Career planning has a significant positive effect on the employability of university students.

### 3.2 The impact of self-concept on employability

According to the Career EDGE model of employability, in addition to career development learning and generic skills, emotional intelligence (EI) is also one of the fundamental first-tier elements for developing employability (Dacre and Sewell, 2007). Emotional intelligence-also referred to as emotional quotient—refers to qualities related to emotion, affect, willpower, and resilience, as well as the ability to reason with emotions and use them to enhance thinking. It includes the capacity to accurately perceive emotions, to generate emotions that facilitate thought, to understand emotions and emotional knowledge, and to regulate emotions reflectively in ways that promote emotional and intellectual growth (Mayer et al., 2004). Emotional intelligence can be improved through reasoning, perception, and control of emotions, as well as understanding emotional experiences. It has a positive correlation with individual achievement—those with high emotional intelligence tend to be more self-motivated, achieve more, attain greater career success, build stronger interpersonal networks, and enjoy a healthier life compared to those with low emotional intelligence (Cooper, 1997). Self-concept is a component of emotional intelligence within the Career EDGE model. Research indicates that in higher education, students’ self-concept is positively related to both academic achievement and future employability (Kertechian, 2025).

Kim et al. (2015) drawing on self-concept theory, demonstrated that self-concept can be a prerequisite for perceived employability. From the combined perspectives of self-concept theory and human capital theory, perceived employability can be understood more comprehensively. In addition, Zhao et al. (2025) argued that self-concept is an important factor in the formation of employability within the school environment, and that the extent to which individuals act on their self-concept influences their future employability in the workplace. Similarly, Ahmid et al. (2023) also confirmed a highly significant positive effect of self-concept on students’ employability-that is, the stronger a student’s self-concept, the higher their employability. A positive self-concept can enhance employability because it enables individuals to adopt a proactive stance in the face of challenges, to better appreciate themselves, and to take feasible, positive steps toward securing employment, thereby improving their employability (Robert and Vandenberghe, 2021).

Accordingly, the following hypothesis is proposed:

*H2*: Self-concept has a significant positive effect on the employability of university students.

### 3.3 The mediating effect of learning attitude between career planning and employability

Learning attitude refers to a learner’s consistent and sustained psychological state and behavioral tendency toward the content they study, encompassing cognitive, emotional, and behavioral components (Tzafilkou et al., 2021). Research has shown that learning attitude has a significant direct positive effect on employability. In other words, students with a positive learning attitude tend to develop stronger employability, whereas those with a negative learning attitude often demonstrate lower employability (Qiu, 2013). Shen et al. (2026) in exploring factors influencing employability, found that students’ learning attitudes can effectively predict certain aspects of their employability. Moreover, Dalrymple et al. (2021) conceptualized employability as a process rather than a fixed outcome, arguing that employability is not a stable state that can be indefinitely maintained, as it depends on various dynamic personal and environmental factors. For instance, employability may be strengthened or weakened by fluctuations in personal factors such as emotions and attitudes, including learning attitude (Healy, 2023). Consistent with this, Fan and Pan (2022) emphasized that during their time at university, students should adjust their learning goals, attitudes, and methods in line with real-world circumstances to continuously enhance their professional knowledge and skills, which will in turn improve their future employability.

According to the Career EDGE model of employability, career planning is part of the fundamental first-tier elements and is one of the key factors for enhancing employability, (Dacre and Sewell, 2007). From the perspective of career psychological capital, Sepahvand et al. (2023) argued that career planning has a positive effect on career psychological capital-a positive psychological state that individuals demonstrate during their growth and development, which includes traits such as self-confidence, optimism, and positivity. A positive learning attitude is an important component of psychological capital; therefore, career planning can positively influence learning attitude. Furthermore, Tong and Gao (2022) argued that career planning guidance can help students clarify their learning goals and development direction. It can also help them form a positive and active learning attitude, reduce aimless learning, and improve their future employability. However, there is still a lack of clear empirical research verifying whether learning attitude mediates the relationship between career planning and employability. Based on the Career EDGE model, this study seeks to test the mediating effect of learning attitude, thereby addressing this empirical gap in the literature.

Accordingly, the following hypotheses are proposed:

*H3*: Career planning has a significant positive effect on the learning attitude of university students.

*H4*: Learning attitude has a significant positive effect on the employability of university students.

*H5*: Learning attitude mediates the relationship between career planning and employability among university students.

### 3.4 The mediating effect of learning attitude between self-concept and employability

According to the Career EDGE model of employability, self-concept is an important component of personality and a determinant of individual attitudes and behaviors (Stuart, 2006). Individuals with a positive self-concept tend to feel confident, adopt a proactive stance toward challenges, and take feasible, constructive steps to prepare for success (Chen et al., 2020). Research has shown that self-concept has a significant positive effect on learning attitude; in other words, the stronger a university student’s self-concept, the more positive their learning attitude tends to be (Lau et al., 2020). Conversely, individuals with a negative self-concept are more likely to envy others, have poor control over their emotions, and perceive themselves as inferior or incompetent. This lack of self-confidence or hesitation in trying new things inevitably affects their learning attitude (Healy, 2023). Furthermore, Hui and Leung (2025) argued that self-concept influences students’ learning attitudes in such a way that those with stronger self-concepts, higher expectations for the future, and greater belief in their own abilities tend to have more positive learning attitudes. Conversely, students with weaker self-concepts often lack confidence in their learning abilities, doubt their capacity to master academic content, and therefore exhibit more negative learning attitudes.

Research has found that university students’ career self-concept has a significant positive effect on their learning attitude. The clearer students perceive their career self-concept, the better their perceived learning attitude, leading to more proactive learning behaviors, enhanced learning outcomes, improved academic performance, and greater employability (Foster, 2026). In addition, self-concept has a clear direct effect on academic performance, learning attitude, and employment outcomes, and it can also indirectly influence future employability through learning attitude (Hsiao, 2009). Similarly, Robert and Vandenberghe (2021) suggested that students with a well-defined self-concept can see their true selves more clearly, better understand their values, interests, and abilities, and thus develop a more positive learning attitude, which translates into stronger employability. Although existing research has examined the relationship between self-concept and learning attitude, few studies have explored in depth the potential mediating role of learning attitude between self-concept and employability. Based on the Career EDGE model, this study further investigates how self-concept influences employability through learning attitude.

Accordingly, the following hypotheses are proposed.

*H6*: Self-concept has a significant positive effect on the learning attitude of university students.

*H7*: Learning attitude mediates the relationship between self-concept and employability among university students.

## 4 Research method

### 4.1 Research framework

**Figure 2:**
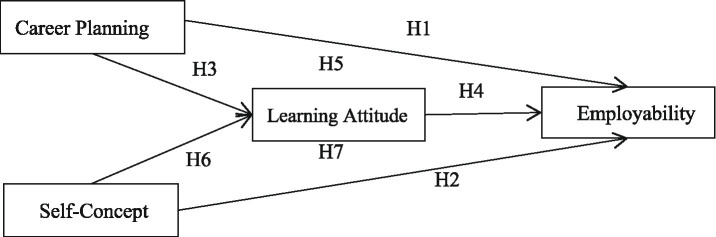
Research framework.

This study is based on the Career EDGE model of employability and focuses on university students in Hebei Province, China. Using SPSS Statistics 27 and AMOS software, it examines the effects of career planning, self-concept, and learning attitude on employability, and tests the mediating role of learning attitude in the relationships between career planning and employability, as well as between self-concept and employability. The research framework is illustrated as follows (see Figure 2).

### 4.2 Research participants and procedure

Hebei Province plays an important role in the Beijing-Tianjin-Hebei coordinated development strategy, with the central government assigning distinct functional roles to each city and leveraging the unique advantages of local resources (Wang et al., 2021). The province has a relatively large number of higher education institutions distributed across a wide area, yet employment levels vary considerably among them (Song and Ren, 2019). The coordinated development of Beijing-Tianjin-Hebei has significantly increased the supply of human resources, intensifying competition among universities in Hebei and increasing employment pressure. In response, the Hebei Provincial Party Committee and the provincial government have consistently adhered to the strategy of prioritizing employment, making every effort to maintain stability in the job market (Wang et al., 2021). At the same time, to enhance university students’ employability and address employment challenges, Hebei has introduced a series of policies specifically targeting graduate employment (Feng et al., 2021). Against this backdrop, this study selected university students in Hebei Province, China, as the research participants and conducted a questionnaire survey.

A pilot survey was first conducted to test whether the scales used in this study were suitable for the sample. Using convenience sampling, 130 university students from one higher education institution were selected for the pilot test. The collected pilot questionnaires underwent reliability, validity, and item analyses, which informed the development of the final questionnaire. For the main survey, convenience sampling was again employed. Participants were drawn from five universities in Hebei Province, including two in Shijiazhuang and one each in Hengshui, Langfang, and Baoding, covering a balanced mix of comprehensive, normal, and science and engineering universities. To avoid overlap with the pilot sample, questionnaires were distributed via the Wenjuanxing online survey platform, where students completed the survey on-site by scanning a QR code. The platform was set to collect participants’ WeChat nickname, gender, and location to prevent duplicate responses. To ensure data quality, student affairs staff from each university were contacted to help organize the sampled students. These staff members explained the purpose and significance of the study to students in class, clarified the instructions for completing the questionnaire, and reminded them to answer carefully. Participation was voluntary, and completion of the questionnaire was taken as implied consent to participate in the research. All participants signed an informed consent form before completing the questionnaire. Their willingness was fully respected, and they were informed that they could refuse to take part in the study or withdraw from it at any time. This study was approved by the Academic Ethics Committee of Dhurakij Pundit University, Thailand.

According to Gorsuch (1983), the minimum sample size for a formal study should be at least 10 times the total number of items in the scale. In this study, the four scales contained a total of 48 items, meaning that at least 480 valid responses were required. Considering the possibility of invalid responses during data collection, 800 questionnaires were distributed to university students from five higher education institutions in Hebei Province, with 160 questionnaires allocated to each institution. After invalid questionnaires with patterned responses, missing answers, or very short completion times being removed, 735 valid questionnaires were obtained, yielding a valid response rate of 91.88%. The final sample met the above sampling standard and was therefore suitable for hypothesis testing and analysis. Among the respondents, 233 were male and 502 were female; 146 were first-year students, 129 were second-year students, 248 were third-year students, and 212 were fourth-year students.

### 4.3 Research instruments

#### 4.3.1 Career Planning Scale

This study adopted the three-item version of Gould’ s (1979) Career Planning Scale as used by Koen et al. (2013), who applied it in educational settings. The scale includes items such as: “I have planned my career,” “I have a strategy to achieve my career goals,” and “I know what I need to do to achieve my career goals,” totaling three items. Responses were measured on a five-point Likert scale ranging from 1 (“strongly disagree”) to 5 (“strongly agree”). Higher total scores indicate better career planning. Reliability analysis of the pilot test showed a Cronbach’s Alpha of 0.836, indicating good internal consistency.

Confirmatory factor analysis (CFA) was conducted using the formal questionnaire, and the results indicated a saturated model. A saturated model refers to a hypothesized model in which the number of estimated parameters exactly equals the number of elements in the covariance matrix. In this case, all parameters have a unique solution, and both the chi-square value and the degrees of freedom are zero. Since the chi-square value is zero, the model is considered just-identified, meaning it will never be rejected and achieves a perfect fit with the data (Wu, 2010). Furthermore, all factor loadings were statistically significant at the *p* < 0.001 level, with standardized factor loadings ranging from 0.797 to 0.854, all exceeding the 0.500 threshold. The composite reliability (CR) of the latent construct was 0.869, meeting the recommended criterion of greater than 0.700 (Hair et al., 1998). The average variance extracted (AVE) was 0.689, exceeding the recommended value of 0.500 (Fornell and Larcker, 1981). These results indicate that the Career Planning Scale demonstrates good convergent validity.

#### 4.3.2 Self-Concept Scale

This study adopted the Self-Concept Scale developed by Hsiao (2009), which measures university students’ self-concept using items drawn from the Higher Education Database. The scale consists of six items. Responses were measured on a five-point Likert scale ranging from 1 (“strongly disagree”) to 5 (“strongly agree”), with reverse-scored items coded accordingly. Reliability analysis of the pilot test yielded a Cronbach’s *α* of 0.886, indicating good internal consistency.

Confirmatory factor analysis (CFA) was conducted using the formal questionnaire to assess model fit and convergent validity. The results showed that *χ*^2^/df = 3.619, which is less than 5; GFI = 0.986 and AGFI = 0.966, both exceeding the 0.900 threshold; SRMR = 0.019 and RMSEA = 0.060, both below the 0.080 standard; NFI = 0.986, RFI = 0.976, CFI = 0.990, and IFI = 0.990, all greater than 0.900; PNFI = 0.591, above the 0.500 criterion; and CN = 382, greater than 200. These results indicate that the self-concept model has good model fit. In addition, all factor loadings were statistically significant at *p* < 0.001, with standardized factor loadings ranging from 0.701 to 0.854, all exceeding the 0.500 benchmark. The composite reliability (CR) of the latent construct was 0.893, surpassing the recommended value of 0.700 (Hair et al., 1998). The average variance extracted (AVE) was 0.583, above the 0.500 threshold (Fornell and Larcker, 1981). These findings indicate that the Self-Concept Scale demonstrates good convergent validity.

#### 4.3.3 Learning Attitude Scale

This study adopted the revised Learning Attitude Scale developed by Xu et al. (2010), which was adapted from the scales of Zhang (1999) and Fennema and Sherman (1976). The scale consists of three dimensions-cognitive attitude, affective attitude, and behavioral attitude-with a total of 27 items. Responses were measured on a five-point Likert scale ranging from 1 (“strongly disagree”) to 5 (“strongly agree”). Higher total scores indicate a more positive learning attitude among university students. During the item analysis of the pilot questionnaire, items 15 to 18 did not meet the item analysis criteria and were therefore removed. The pilot test yielded a Cronbach’s *α* coefficient of 0.936, indicating high reliability.

Confirmatory factor analysis (CFA) was conducted using the formal questionnaire to assess model fit and convergent validity. The results showed that *χ*^2^/df = 2.333, less than the recommended threshold of 5; GFI = 0.947 and AGFI = 0.935, both above 0.900; SRMR = 0.030 and RMSEA = 0.043, both below 0.080; NFI = 0.942, RFI = 0.936, CFI = 0.966, and IFI = 0.966, all exceeding 0.900; PNFI = 0.846 and PGFI = 0.779, both greater than 0.500; and CN = 365, greater than 200. These results indicate that the learning attitude model has good model fit. All factor loadings were statistically significant at *p* < 0.001, with standardized factor loadings ranging from 0.679 to 0.823, all above the 0.500 benchmark. The composite reliability (CR) values for the latent variables were: cognitive attitude = 0.912, affective attitude = 0.848, and behavioral attitude = 0.912, all exceeding the recommended criterion of 0.700 (Hair et al., 1998). The average variance extracted (AVE) values were: cognitive attitude = 0.535, affective attitude = 0.528, and behavioral attitude = 0.536, all above the 0.500 threshold (Fornell and Larcker, 1981). These results indicate that the Learning Attitude Scale demonstrates good convergent validity.

#### 4.3.4 Employability scale

This study adopted the Employability Scale revised by Cheng and Dong (2016), which consists of four factors: basic competence, social practice ability, professional ethics, and job-seeking ability, with a total of 16 items. Responses were measured on a five-point Likert scale ranging from 1 (“very poor”) to 5 (“very strong”). Higher total scores indicate stronger employability. The scale’s Cronbach’s *α* coefficient was 0.881, indicating good reliability.

Confirmatory factor analysis (CFA) was conducted using the formal questionnaire to assess model fit and convergent validity. The results showed that *χ*^2^/df = 3.300, less than the recommended threshold of 5; GFI = 0.951 and AGFI = 0.934, both above 0.900; SRMR = 0.032 and RMSEA = 0.056, both below 0.080; NFI = 0.947, RFI = 0.936, CFI = 0.962, and IFI = 0.962, all exceeding 0.900; PNFI = 0.789 and PGFI = 0.699, both greater than 0.500; and CN = 277, greater than 200. These indices indicate that the employability model has good model fit. All factor loadings were statistically significant at *p* < 0.001, with standardized factor loadings ranging from 0.689 to 0.872, all above the 0.500 benchmark. The composite reliability (CR) values for the latent variables were as follows: basic competence = 0.806, social practice ability = 0.896, professional ethics = 0.840, and job-seeking ability = 0.856, all exceeding the recommended criterion of 0.700 (Hair et al., 1998). The average variance extracted (AVE) values were as follows: basic competence = 0.676, social practice ability = 0.552, professional ethics = 0.569, and job-seeking ability = 0.665, all above the 0.500 threshold (Fornell and Larcker, 1981). These results indicate that the Employability Scale demonstrates good convergent validity.

## 5 Research results

### 5.1 Common method bias test

Since this study’s questionnaire included more than two variables and all variables were answered by the same participants, there was a possibility of common method bias due to personal or psychological factors. To reduce this risk, the study followed the recommendation of Zhou and Long (2004) and applied Harman’s single-factor test through exploratory factor analysis to detect common method variance. All items from the formal questionnaire were entered into SPSS for factor analysis, and the variance explained by the first unrotated principal component was examined. If the variance explained by the first factor is less than 50%, it suggests that there is no serious common method bias.

As shown in Table 1, the analysis produced nine factors with eigenvalues greater than 1, and the first factor accounted for 31.499% of the variance, which is below the 50% threshold. Therefore, there is no significant common method variance in the formal questionnaire sample, and further data analysis can proceed.

**Table 1:** Analysis of common method bias.

Factor	Eigenvalue (≥1)	Variance explained (%)	Cumulative variance explained (%)
1	15.120	31.499	31.499
2	4.368	9.099	40.598
3	2.567	5.347	45.946
4	2.237	4.660	50.606
5	1.622	3.380	53.986
6	1.551	3.232	57.217
7	1.534	3.197	60.414
8	1.185	2.470	62.883
9	1.063	2.214	65.098

In addition, common method bias was tested by comparing the model fit of the confirmatory factor analysis (CFA) for the multi-factor model and the single-factor model. The results showed that the chi-square value of the multi-factor model (*χ*^2^ = 5066.417, df = 1,074) was significantly lower than that of the single-factor model (Δ*χ*^2^ = 9880.710, Δdf = 1,080, *p* < 0.001). This indicates that there was a significant difference between the two models. Therefore, the data did not show serious common method bias (Richardson et al., 2009), and further data analysis could be conducted.

### 5.2 Correlation analysis

Pearson correlation analysis was conducted to examine the relationships among the variables. As shown in Table 2, the correlation coefficients ranged from 0.361 to 0.520, and all correlations were significantly positive (*p* < 0.001). This indicates that there are significant positive relationships among the variables. Furthermore, since none of the correlation coefficients exceeded 0.800, there is no concern of multicollinearity (Maruyama, 1998). Therefore, it is appropriate to proceed with the subsequent overall model validation analysis.

**Table 2:** Results of correlation analysis.

Variable	Mean	SD	Career planning	Self-concept	Learning attitude	Employability
Career Planning	3.470	0.856	**0.831** ^**a**^			
Self-Concept	3.732	0.804	0.361***	**0.764** ^**a**^		
Learning Attitude	3.660	0.561	0.465***	0.396***	**0.802** ^**a**^	
Employability	3.747	0.527	0.496***	0.494***	0.520***	**0.753** ^**a**^

*p* < 0.001. ^a^The bold values on the main diagonal represent the square roots of the AVE values for each scale.

For the correlations among the constructs, as shown in Table 2, Hair et al. (1998) suggested that the correlation coefficient between two different constructs should be lower than the square root of the average variance extracted (AVE) for each construct. The square root of the AVE for each construct met this standard. Therefore, the discriminant validity was good.

### 5.3 Path analysis of the overall model

Based on the research hypotheses, a path analysis was conducted to examine the relationships among career planning, self-concept, learning attitude, and employability among university students. The overall model fit was first evaluated. Following Schumacker and Lomax (2004), this study assessed the model using three types of fit indices: absolute fit, parsimonious fit, and incremental fit. The evaluation criteria were as follows: *χ*^2^/df less than 5 (Schumacker and Lomax, 2004); GFI, IFI, NFI, CFI, and TLI greater than 0.900; and RMSEA less than 0.080 (Hair et al., 1998). As shown in Table 3, the results indicate that *χ*^2^/df = 2.571, which is less than 5; GFI = 0.959 and AGFI = 0.944, both greater than 0.900; RMSEA = 0.046, less than 0.080; NFI = 0.956, RFI = 0.947, CFI = 0.973, and IFI = 0.973, all greater than 0.900; PNFI = 0.789 and PGFI = 0.698, both greater than 0.500; and CN = 356, exceeding 200. These results demonstrate that the model has a good overall fit.

**Table 3:** Fit indices for the overall model.

Fit category	Index	Criterion	Value	Model fit
Absolute fit	*χ* ^2^ */df*	<5	2.571	Good fit
	GFI	>0.900	0.959	Good fit
	AGFI	>0.900	0.944	Good fit
	RMSEA	<0.080	0.046	Good fit
Incremental fit	NFI	>0.900	0.956	Good fit
	RFI	>0.900	0.947	Good fit
	CFI	>0.900	0.973	Good fit
	IFI	>0.900	0.973	Good fit
Parsimonious fit	PNFI	>0.500	0.789	Good fit
	PGFI	>0.500	0.698	Good fit
	CN	>200	356	Good fit

#### 5.3.1 Direct effects

**Figure 3:**
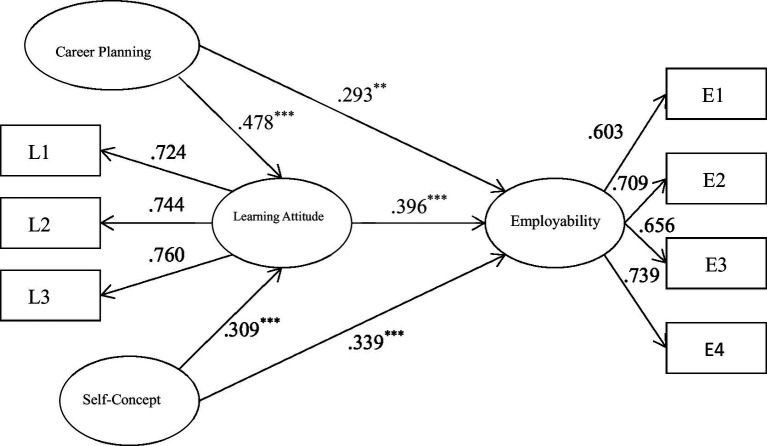
Mediation model path diagram.

As shown in Table 4 and Figure 3, career planning had a significant positive effect on employability, with a path coefficient of 0.293 (*p* < 0.001) and a 95% confidence interval of [0.199, 0.387], which does not include zero. Self-concept had a significant positive effect on employability, with a path coefficient of 0.339 (*p* < 0.001) and a 95% confidence interval of [0.253, 0.420], also excluding zero. Career planning had a significant positive effect on learning attitude, with a path coefficient of 0.478 (*p* < 0.001) and a 95% confidence interval of [0.392, 0.565]. Learning attitude had a significant positive effect on employability, with a path coefficient of 0.396 (*p* < 0.001) and a 95% confidence interval of [0.298, 0.498]. Self-concept also had a significant positive effect on learning attitude, with a path coefficient of 0.309 (*p* < 0.001) and a 95% confidence interval of [0.216, 0.398]. These results indicate that all five direct effects are significant, suggesting that career planning, self-concept, and learning attitude enhance university students’ employability, while career planning and self-concept also influence their learning attitude. Therefore, hypotheses H1, H2, H3, H4, and H6 are all supported.

**Table 4:** Bootstrap test and path analysis.

Path	Effect	*p*	95% CI
Direct effects			
Career planning → employability	0.293	0.000	[0.199, 0.387]
Self-concept → employability	0.339	0.000	[0.253, 0.420]
Career planning → learning attitude	0.478	0.000	[0.392, 0.565]
Self-concept → learning attitude	0.309	0.000	[0.216, 0.398]
Learning attitude → employability	0.396	0.000	[0.298, 0.498]
Indirect effects			
Career planning → learning attitude → employability	0.189	0.000	[0.137, 0.258]
Self-concept → learning attitude → employability	0.122	0.000	[0.080, 0.176]
Total effects			
Career planning → employability	0.482	0.000	[0.397, 0.565]
Self-concept → employability	0.461	0.000	[0.372, 0.544]

CI, confidence interval. All effects are significant at *p* < 0.001 based on 5,000 bootstrap samples.

To further examine the mediating effect of learning attitude between career planning and employability, as well as between self-concept and employability, the bootstrap method was applied following the recommendations of Hayes (2013), with 5,000 resamples.

#### 5.3.2 Indirect effects

As shown in Table 4 and Figure 3, the indirect effect of learning attitude between career planning and employability was 0.189 (*p* < 0.001), with a 95% confidence interval of [0.137, 0.258], which does not include zero. This indicates that the indirect effect is significant, suggesting that career planning can indirectly influence employability through learning attitude, thereby confirming the partial mediating effect of learning attitude. Thus, hypothesis H5 is supported. Similarly, the indirect effect of learning attitude between self-concept and employability was 0.122 (*p* < 0.001), with a 95% confidence interval of [0.080, 0.176], also excluding zero. This indicates that the indirect effect is significant, suggesting that self-concept can enhance employability through the strengthening of learning attitude, confirming the partial mediating effect of learning attitude. In other words, self-concept not only has a direct positive effect on employability, but may also indirectly influence employability via learning attitude. Therefore, hypothesis H7 is supported.

## 6 Discussion

### 6.1 Career planning’s direct effect on employability

The results indicate that career planning has a significant positive effect on the employability of university students. Thus, Hypothesis H1 is supported. This finding is consistent with previous studies (Chughtai, 2019; Clements and Kamau, 2018), suggesting that career planning plays an active role in enhancing students’ employability and is an important influencing factor. According to the Career EDGE model, career planning belongs to the dimension of “career development learning” at the first level of the model. It also partly overlaps with planning, coordination, organization, and management skills in “generic skills” (Dacre and Sewell, 2007). The model clearly states that employability development begins with the accumulation of basic elements at the first level. Among these elements, career development learning is regarded as an important condition for students to find satisfactory jobs. The core of career development learning is to help students improve their self-awareness, clarify their interests and personal characteristics, and make careful career decisions based on this understanding. Career planning is the specific behavioral expression of this process. Through career exploration, career goal setting, action path planning, evaluation, and adjustment, individuals gradually clarify their career development direction. This goal-oriented behavior can effectively reduce students’ blindness in employment preparation and improve their accurate understanding of the job market. As a result, it can directly enhance their perceived employability (Salisu et al., 2025). Furthermore, career planning can enhance students’ self-awareness. Self-awareness requires students to use various methods-such as career interest tests, assessments of career values, and evaluations of professional skills-to gain a clearer understanding of themselves. This process helps students better define their future career development direction, choose occupations that match both their interests and capabilities, and make more rational career choices. It also reduces the influence of conformity and impulsive decision-making on future employment, thus improving employability (Guo, 2021). In summary, career planning can positively predict employability. By engaging in career planning, students gain a clearer understanding of their career development goals, enhance their self-awareness, and make rational decisions, ultimately contributing to improved employability. Therefore, this study introduced the model to Chinese university students and tested the positive predictive effect of career planning on employability in an Eastern educational and cultural context. In this way, the study enriches the cross-cultural applicability of the model.

In summary, career development learning is a basic element at the first level of the Career EDGE model. Universities should include it in general education or compulsory courses. This can help students build career exploration awareness from the early years of university, rather than receiving short-term job-seeking guidance only when they are close to graduation.

### 6.2 Self-concept’s direct effect on employability

The results show that self-concept has a significant positive effect on the employability of university students, confirming Hypothesis H2. This finding is consistent with previous studies (Zhao et al., 2025; Ahmid et al., 2023), indicating that a strong self-concept can contribute to the enhancement of employability. In other words, when university students possess a positive self-concept, it fosters greater confidence, self-efficacy, and a proactive approach to career development, all of which strengthen their employability. According to the Career EDGE model, self-concept belongs to the dimension of emotional intelligence. Emotional intelligence includes the ability to perceive emotions, understand emotional knowledge, and regulate emotions through reflection in order to support intellectual growth. As an individual’s overall evaluation of the self, self-concept directly affects how a person responds emotionally to employment-related situations. People with a high level of emotional intelligence can gain more self-motivation and achieve more. In the employment context, students with a positive self-concept are more likely to make positive attributions and regulate their emotions when they face job-seeking setbacks. This helps them maintain continuous action. Such ability to use emotional resources directly improves their employability.

Students with a positive self-concept tend to have a clear understanding of themselves and their capabilities, and they are more likely to engage in proactive employment behaviors, such as actively acquiring new knowledge and skills and seeking job opportunities. Conversely, those with a negative self-concept may exhibit avoidant or passive job-seeking behaviors, lacking the confidence and courage to act on their abilities, which results in hesitation and passive responses, ultimately diminishing their employability. In addition, students with a positive self-concept are generally better equipped to manage stress and overcome difficulties. They tend to have stronger self-awareness and emotional stability, which can reduce the anxiety caused by job-search pressures. In contrast, students with a negative self-concept may experience anxiety, low self-esteem, and timidity when facing employment difficulties, making it harder to cope effectively with future challenges, thus lowering their employability (Kim et al., 2015). In summary, self-concept influences students’ employment motivation, job-seeking behaviors, and psychological readiness, thereby affecting their employability. Enhancing university students’ employability requires giving due attention to the cultivation of their self-concept. Therefore, this study distinguishes three influence paths: cognitive integration, emotional mobilization, and reflective growth. This goes beyond the limitation of previous studies that only reported the correlation between self-concept and employability. It provides a more complete theoretical framework for understanding why self-concept can improve employability.

In summary, self-concept is a basic psychological resource for employability development. Universities should help students build a clear, positive, and stable understanding of themselves through career guidance, psychological education, and personalized development support, rather than only focusing on surface-level training in job-seeking skills.

### 6.3 The mediating effect of learning attitude between career planning and employability

The findings show that learning attitude mediates the relationship between career planning and employability among university students. Hypotheses H3, H4, and H5 are supported. This result is consistent with the findings of Sepahvand et al. (2023), indicating that career planning can indirectly influence employability through learning attitude. According to the Career EDGE model, career planning, as a first-level behavioral practice, includes activities such as career exploration, goal setting, and action planning. However, these activities do not directly mean that employability has improved. Individuals need to continuously reflect on and evaluate these planning behaviors and their outcomes before they can truly turn them into internal ability resources. Learning attitude is a clear expression of this reflective process. A positive learning attitude means that individuals approach learning activities with an open, active, and serious mindset. It helps them deeply process their learning experiences and transform the accumulated behaviors of career planning into a real improvement in employability.

Career planning allows students to establish a correct outlook on life. By clarifying learning goals and developing a comprehensive plan suited to their personal circumstances, students are motivated to study diligently in order to realize their life plans and values, acquire more skills needed for the future, and ultimately enhance their employability. Through career planning, students can develop effective learning strategies, methods, and emotional regulation skills, thereby improving their learning character. This leads to a more active learning attitude, which promotes personal growth and strengthens employability (Wang et al., 2023). Therefore, this study clarifies the role of learning attitude as a mediating variable. It answers the theoretical question of how career planning influences employability. In this way, it deepens the theoretical understanding of career planning research.

In summary, for university students, the practical meaning of this result is that it identifies a key point for self-improvement. In the face of a difficult employment situation, anxiety about external job competition is often not very effective. In contrast, setting career plans as early as possible and using them to adjust one’s current learning attitude is a more reliable way to deal with the problem. A good learning attitude is not only a guarantee of academic success, but also a foundation for building long-term employability.

### 6.4 The mediating effect of learning attitude between self-concept and employability

The results indicate that learning attitude mediates the relationship between self-concept and employability among university students. This finding is consistent with previous research (Hsiao, 2009; Lau et al., 2020) and supports Hypotheses H6 and H7, suggesting that self-concept can indirectly influence employability through learning attitude. In other words, students’ self-concept shapes their learning attitude during university, which in turn affects their employability. In the Career EDGE model, self-concept belongs to the dimension of emotional intelligence. Individuals with high emotional intelligence can gain more self-motivation and achieve more. As an individual’s overall evaluation of the self, self-concept directly affects their emotional experience and emotion regulation ability in learning situations. Specifically, students with a positive self-concept are more likely to make positive attributions when they face learning difficulties, such as “I can overcome this through effort.” This helps them maintain or even strengthen a positive learning attitude. In contrast, students with a negative self-concept are more likely to develop negative learning attitudes, such as learning burnout and avoidance of difficulties. The learning attitude shaped by self-concept then becomes an important psychological resource that affects the improvement of employability.

In addition, when students have a clear self-concept, they are more certain about their choices, better able to see their true selves, and more aware of their values, interests, and abilities. This self-awareness fosters clear intrinsic motivation, which translates into maintaining a positive learning attitude. Such an attitude manifests in constructive learning behaviors that ultimately enhance employability (Foster, 2026). By building a positive self-concept and strong core self-evaluations, students become more confident and proactive when facing employment challenges. This proactive mindset encourages them to persevere in their learning goals, working hard even when confronted with academic or life difficulties. This persistence and effort ultimately contribute to improving their employability and competitiveness. Based on the above analysis, this study starts from the psychological mechanism of self-concept, learning attitude, and employability. It reveals the dynamic psychological process through which employability is formed. It also provides a more integrated analytical framework for future research.

Therefore, in practice, attention should be paid to the hidden mechanism of “internal motivation transformation.” The development of self-concept should be closely combined with the guidance of learning attitude. By helping students build a positive self-identity, universities can stimulate their internal motivation to actively engage in knowledge learning, skill development, and career planning. This can build a sustainable foundation for employability. This shows that effective employability development should not only focus on skills training. It should also consider the strengthening of confidence and the reshaping of attitudes. Only in this way can students’ short-term job-seeking performance and long-term career growth be improved together.

In summary, this study mainly focused on the effects of self-concept and career planning on employability. However, from the dynamic view of career development, there may be an internal interaction mechanism between these two predictors. According to social cognitive career theory, self-concept, as a core part of an individual’s self-schema, may promote more active and specific career planning behavior by influencing self-efficacy. In other words, career planning may partly mediate the effect of self-concept on employability. Although the cross-sectional data model in this study mainly tested the parallel predictive effects of these two variables, it is still important to recognize and discuss the internal logical chain of “self-cognition-planning behavior.” This can help us better understand the psychological mechanism of career development. It is also a direction that future research should further examine.

## 7 Conclusion and implications

### 7.1 Conclusion

Using SPSS and AMOS, this study surveyed Chinese university students to analyze the effects of career planning and self-concept on employability, with a particular focus on examining the mediating role of learning attitude. The main findings are as follows:

#### 7.1.1 Direct effects

Career planning, self-concept, and learning attitude all have significant direct effects on employability. In addition, career planning and self-concept were found to positively predict learning attitude.

#### 7.1.2 Indirect effects

This study further explored the mechanisms through which career planning and self-concept influence employability. The results reveal that career planning positively predicts employability indirectly through the mediating role of learning attitude. Similarly, self-concept also exerts an indirect positive effect on employability via learning attitude.

### 7.2 Implications

This study holds important theoretical and practical implications.

#### 7.2.1 Theoretical implications

First, by integrating career planning, self-concept, learning attitude, and employability into a single theoretical model, this study reflects the relationships and underlying mechanisms among these variables. The findings provide a theoretical basis for enhancing university students’ employability and further enrich the body of theory related to each variable. In particular, learning attitude-conceptualized as a psychological disposition-was shown to mediate the effects of career planning and self-concept on employability. This finding not only offers new empirical support for the Career EDGE model of employability, but also provides a theoretical foundation for better understanding the internal mechanisms influencing students’ employability. Second, while employability has received increasing policy attention in China, research on university students’ employability by Chinese scholars began relatively late. There remains a lack of employability theories and empirical studies with strong local relevance, as well as an absence of broadly applicable theoretical frameworks. Building on the existing literature, this study explores the mechanisms through which career planning, self-concept, and learning attitude influence employability. The results help address existing research gaps and test the applicability of the Career EDGE model in the Chinese context, thereby filling an important theoretical void. Third, much of the current research on employability in China is theoretical in nature, with fewer empirical studies. Existing studies on influencing factors often rely on subjective judgments and lack empirical investigation and data support. By examining the mechanisms linking career planning, self-concept, and learning attitude to employability, this study supplements empirical research on Chinese university students’ employability. It also provides objective, data-driven evidence for understanding the factors that influence employability.

#### 7.2.2 Practical implications

Based on the findings of this study, career planning, self-concept, and learning attitude all have significant positive effects on employability, providing feasible pathways to enhance university students’ employability. First, strengthen career planning education for university students. Universities should integrate career planning into general curricula, providing systematic guidance to help students plan their career paths in advance. This should follow the objective rules of students’ growth and development, tailoring guidance to different stages, levels, and individual characteristics. By aligning career design guidance with students’ developmental needs, institutions can lay a strong foundation for improving employability. Second, cultivate positive learning attitudes to form the intrinsic driving force of employability. Universities should create a positive learning atmosphere to stimulate students’ intrinsic motivation. This can be achieved by establishing learning communities, such as professional study groups, reading clubs, and research interest societies, to reduce feelings of isolation in learning. Additionally, providing students with guidance on effective learning strategies can help them gradually develop an attitude of proactive exploration, responsibility, and continuous growth, thereby laying a solid foundation for employability enhancement. Finally, build growth platforms to foster the development of a positive self-concept. Universities should adopt a student-centered approach, paying attention to students’ feelings, perceptions, and cognition, and promoting deeper self-understanding and reconstruction of self-concept. For example, offering platforms and contexts for learning and growth can allow students to construct interactions between self and society through group engagement, facilitating comprehensive self-development. Educators should pay particular attention to students’ inner worlds, listen to their voices, understand their needs, and help them build a positive self-concept. This includes enhancing students’ emotional regulation abilities and focusing on challenges they face, such as failure or setbacks, by guiding them to develop appropriate self-attribution skills. Furthermore, psychological training camps, group discussions, and other experiential activities can help students understand themselves from multiple perspectives and levels, improving self-awareness and, in turn, strengthening employability.

## 8 Limitations and future research directions

First, this study primarily employed a questionnaire survey method. While this method offers flexibility and convenience, the use of self-reported measures by students may introduce bias, as respondents may conceal their shortcomings during the survey process. As a result, the data may contain slight deviations. To gain deeper insights into the factors influencing employability, future studies could incorporate in-depth interviews with university students to better capture their genuine intentions and explore the multiple factors that affect employability.

Second, the sample for this study was drawn from students at five universities in Hebei Province, China. This sampling approach has a certain degree of regional concentration and may not fully represent the national situation, although it does ensure that the findings are representative of Hebei Province. Future research could expand the geographic scope and coverage of the sample to improve the generalizability of the results. In addition, in economies with higher labor market flexibility or more developed social security systems, this structural conflict may be less serious, or it may exist in different forms. Cultural contexts, such as collectivism and individualism, may also moderate how individuals respond to these structural pressures.

In addition, an important limitation of this study is the serious imbalance in the gender distribution of the sample, with female university students accounting for a high proportion of the respondents. Previous studies have shown that gender may affect individuals’ self-concept and the mechanism through which learning attitude influences employability. Therefore, the current results may mainly reflect the experience pattern of female university students. In future research, gender can be included in the analytical framework as a moderating variable. Researchers can also use different sampling methods to make the gender ratio of the sample more balanced. This would help improve the external validity of the research findings. At the same time, based on the principle of theory-driven approach, this study did not include demographic variables such as gender, grade, and major as control variables in the structural equation model, which may have to some extent overlooked their potential impact on the relationships among the core variables. Future research could use larger samples to further examine the moderating or controlling effects of demographic variables.

Third, while examining the factors influencing employability, this study focused primarily on the individual-level perceptions of students, which may carry a degree of subjectivity. Future research could integrate school-level, teacher-level, and individual-level factors, employing cross-level analytical methods to more comprehensively examine the determinants of employability. In addition, the recommendations and strategies proposed could be applied in university student management practices, allowing for practical evaluation of their effectiveness in enhancing employability and thereby broadening and deepening the scope of research.

Fourth, the results indicate that career planning and self-concept have significant positive effects on employability. However, when learning attitude was included as a mediating variable, the path coefficients for the effects of career planning on employability and self-concept on employability decreased. This suggests that learning attitude plays a partial mediating role in both relationships and implies the possible existence of other mediating variables. Future studies could further investigate alternative mediators or examine potential moderating variables to refine and extend the research model.

Fifth, the construct of career planning was measured with only three items. Although this scale has been used in previous studies and has reported acceptable internal consistency reliability, the small number of items may not fully cover all aspects of this construct. This may place some limits on content validity. Therefore, when interpreting the related results, we avoid making overly broad conclusions. Future research should use a more complete multidimensional scale to measure career planning, so as to improve the representativeness of the construct and the validity of measurement.

## Data Availability

The raw data supporting the conclusions of this article will be made available from the corresponding author, without undue reservation.
